# Non-invasive Diagnostics in the Focus: The Importance of Salivary Biomarkers in Detecting Oral Cancer

**DOI:** 10.7759/cureus.97626

**Published:** 2025-11-24

**Authors:** Anam Khan, Jahan Elavia, Reema Srichand, Swapnali Mhatre, Deepak Sharma, Uttam Shetty

**Affiliations:** 1 Prosthodontics, Bharati Vidyapeeth Dental College and Hospital, Navi Mumbai, IND; 2 Prosthodontics, Crown and Bridge, and Implantology, Bharati Vidyapeeth Dental College and Hospital, Navi Mumbai, IND; 3 Endodontics, Bharati Vidyapeeth Dental College and Hospital, Navi Mumbai, IND

**Keywords:** early detection, non-invasive diagnostics, oral cancer, salivaomics, salivary biomarkers

## Abstract

Oral squamous cell carcinoma (OSCC) is a global health challenge, often diagnosed at advanced stages due to the invasiveness and limited accessibility of current methods like tissue biopsies. Salivary biomarkers have emerged as a transformative alternative, offering a non-invasive, cost-effective, and patient-friendly approach for early detection, prognosis, and treatment monitoring. This review highlights the mechanistic roles of salivary biomolecules, including microRNAs (miRNAs) (e.g., miR-31, miR-200a), proteins (interleukin-8 (IL-8), matrix metalloproteinase-9 (MMP-9)), circulating tumor DNA (ctDNA), and metabolites, in OSCC pathogenesis. We discuss advancements in salivaomics (genomics, proteomics, metabolomics) and technologies (biosensors, AI-driven multi-marker panels) that enhance diagnostic accuracy. Published data from validation studies indicate that several salivary biomarkers demonstrate strong diagnostic potential for OSCC detection. Despite ongoing advances, significant challenges such as biomarker variability, lack of standardized collection protocols, and the need for robust clinical validation currently limit the widespread adoption of salivary diagnostics in OSCC. Future research is focusing on integrated multi-omics strategies, the development of wearable real-time monitoring devices, and establishing regulatory frameworks to better facilitate clinical implementation. Addressing these issues may improve the reliability and accessibility of saliva-based diagnostics for OSCC, particularly in low-resource and high-risk populations.

## Introduction and background

Oral cancer, primarily oral squamous cell carcinoma (OSCC), is a significant global public health issue, responsible for a large burden of cancer morbidity and mortality. OSCC is the sixth most common cancer worldwide, with approximately 275,000 new cases reported annually [1]. Although it is highly preventable and curable when detected at an early stage, OSCC is primarily diagnosed late, with poor outcomes and low survival. The conventional diagnostic methods, visual examination, tissue biopsy, histopathology, and imaging, are still the gold standard [2]. However, the methods are invasive, costly, and not readily available in low-resource settings, and therefore not ideal for widespread application [3]. Moreover, OSCC progresses without any symptoms at its initial phase, and hence it gets diagnosed late, and the cure is not effective. Therefore, other diagnostic tools that are low-cost, non-invasive, and capable of detecting cancer at its initial phase are urgently needed.

Cancer diagnosis and detection have been revolutionized in recent years through the power of biomarker-based diagnosis, providing new promise for risk assessment and detection. Biomarkers, including genetic, epigenetic, proteomic, and metabolic markers, hold the promise of illuminating the progression of the disease and the response to treatment [4]. We know a great deal about markers in the bloodstream, and attention has now been directed to saliva as a diagnostic vehicle due to its unique biochemical properties, ease of sampling, and proximity to oral tumors. Saliva sampling is painless, not invasive, and does not require trained medical personnel [5] in comparison to tissue biopsy or blood sampling, providing the potential to be used as a screening and detection method on a large scale [6]. Recent studies using 'Omics' technologies (genomics, proteomics) confirm that saliva contains reliable biomarkers for early cancer detection [1]. Omics technologies, including genomics, transcriptomics, proteomics, and metabolomics, provide high-throughput platforms to study large-scale molecular changes associated with OSCC. These approaches enable comprehensive profiling of DNA mutations, RNA expression patterns, protein networks, and metabolic alterations. When combined, these layers form the foundation of ‘salivaomics,’ a multi-dimensional analytical framework that significantly enhances the detection accuracy of salivary biomarkers by capturing the complex biology of tumor development. As research in salivary diagnostics progresses, technological advances in biosensors have enabled more rapid and sensitive detection of these biomarkers. Furthermore, the integration of artificial intelligence (AI) and machine learning (ML) now allows for the analysis of complex biomarker data, supporting improved diagnostic accuracy and clinical decision-making.

Saliva is a biofluid rich in many proteins, DNA, RNA, microRNAs (miRNAs), cytokines, metabolites, and exosomes that all have the potential to be oral cancer progression and development markers [7]. Salivary omics technologies, including transcriptomics, proteomics, metabolomics, and microbiome analysis, have matured to the level where an unprecedented number of promising biomarkers have been identified [6]. Of significance, miRNAs have been shown to be key regulators of cancer processes with central roles in tumor growth, cell death, invasion, and metastasis [8]. Certain salivary miRNA signatures have been shown to be highly sensitive and specific in distinguishing between OSCC and benign lesions and thus are excellent candidates for the screening and monitoring of the disease at an early phase. Similarly, tumor-derived extracellular vesicles (EVs) and exosomes in saliva transfer cancer-associated molecules that reflect the tumor environment with high diagnostic potential [4].

Besides molecular markers, novel biosensor technologies, point-of-care diagnostic tools, and liquid biopsy systems have significantly enhanced saliva diagnosis. Incorporating lab-on-a-chip devices, electrochemical sensors, and artificial intelligence-aided analysis of biomarkers into the clinic holds enormous potential for oral cancer detection in real time, at the chairside. Such newer technologies allow high-throughput, low-cost, and quick screening, particularly in community and low-resource settings where conventional diagnostic centers may not be readily accessible. Despite these promising advances, challenges to the integration of salivary biomarkers into daily clinical practice remain. Challenges in standardizing the validation of the biomarkers, managing salivary composition variability between subjects, and the performance of large-scale clinical trials remain to be overcome before widespread application. In addition, advances in AI are assisting in the generation of multi-marker panels and predictive models, which may improve the sensitivity and reproducibility of diagnostic systems based on salivary biomarkers. Despite these technological advances, the field still faces multiple challenges, such as biological variability in biomarkers and the need for clinical validation and standardization. Given these considerations, the current review aims to provide a comprehensive overview by discussing the mechanistic function of salivary biomarkers, assessing their clinical utility, and outlining future directions for research and implementation (Table 1).

**Table 1:** Comparison of saliva-based diagnostics and tissue biopsy across key clinical parameters.

Parameter	Saliva-based diagnostics	Tissue biopsy
Invasiveness	Non-invasive	Highly invasive
Patient comfort	High comfort, painless	Painful, causes anxiety
Sample collection	One to two minutes	20-40 minutes
Cost of procedure	Low cost	High cost
Turnaround time	Rapid	Slow
Risk of complications	None	Bleeding, infection, delayed wound healing
Standardization level	Emerging; still being optimized	Well-established
Ability to monitor treatment response	Excellent, can repeat frequently	Limited; repeated biopsies not feasible
Accessibility in low-resource settings	Highly accessible	Limited accessibility

## Review

This narrative review was developed using searches performed on PubMed, Scopus, Web of Science, and Google Scholar. Keywords included ‘salivary biomarkers,’ ‘salivaomics,’ ‘oral squamous cell carcinoma,’ ‘OSCC detection,’ ‘miRNA saliva,’ and ‘non-invasive cancer diagnostics.’ Articles published between 2010 and 2025 were included. Priority was given to original studies, meta-analyses, and high-quality narrative reviews. Since this is not a systematic review, no Preferred Reporting Items for Systematic reviews and Meta-Analyses (PRISMA) flowchart or formal risk-of-bias assessment was conducted.

Saliva has emerged as a key diagnostic biofluid with its low-cost, easily accessible, and non-invasive characteristics. In recent years, saliva-based diagnosis has achieved substantial progress, particularly in oral cancer detection and tracking, a condition that has been a serious public health concern worldwide. The oral cancer incidence continues to rise due to delayed diagnosis, with a considerable decrease in survival rates. The lack of effective minimally invasive diagnosis has necessitated alternative biomarker-based strategies, and saliva has emerged as a promising biofluid to detect oral cancer due to its high content of molecules. The salivaomics discipline, which includes genomic, transcriptomic, proteomic, metabolomic, microbiomic, and exosomal analyses, has discovered that saliva carries a rich repertoire of biomarkers that can serve as markers of oral cancer development, prognosis, and therapeutic response [9]. Because saliva is in direct contact with the oral cavity, it provides an ideal medium to detect oral cancer-related molecular changes and thus allows early diagnosis, monitoring of the disease, and therapeutic evaluation. Salivary diagnostics is now being envisioned as a precision medicine game-changer that can make targeted therapies and customized treatment regimens possible through biomarker signatures [10].

Saliva: composition, functions, and its biological significance

The major (parotid, submandibular, and sublingual) and minor salivary glands secrete essential biomolecules, which constitute the rest of the amount of saliva, a very dynamic and bioactive fluid that is 99% water. Saliva, secreted by these glands, is crucial for maintaining general and dental health. Saliva is not a biochemically inactive passive fluid, but a biochemically active substance. It consists of an enormous variety of constituents, including proteins, enzymes, lipids, nucleic acids, and metabolites, all of which play significant roles in physiological as well as pathological events [11]. Saliva contains significant electrolytes like sodium, potassium, chloride, calcium, bicarbonate, and phosphate that are involved in pH balance and oral tissue integrity. Saliva also transports biologically active proteins and peptides, such as amylase, mucins, histatins, cystatins, and lactoferrin, which are involved in digestion, tissue protection, and combating infections. All these constituents play a role in the prevention of disease, digestion, and wound healing.

Salivary enzymes play a vital part in defence and digestion. Salivary enzymes such as proteases, lipases, and peroxidases commence biochemical reactions that will help to ensure oral and overall well-being. Nucleic acids (RNA and DNA), lipids, and many metabolites of the saliva are representative of cellular metabolism and disease conditions. Salivary enzymes, such as proteases, lipases, and peroxidases, commence biochemical reactions that will help to ensure oral and overall well-being. Nucleic acids (RNA and DNA), lipids, and many metabolites of the saliva are representative of cellular metabolism and disease conditions. One of the most fascinating aspects of it is extracellular vesicles like exosomes, which carry biomolecules related to tumors and are hence of research interest in cancer biomarker research. Salivary enzymes have an important role to play in both defence and digestion. Salivary enzymes like proteases, lipases, and peroxidases initiate biochemical reactions that assist in maintaining oral and general health. Nucleic acids (RNA and DNA), lipids, and several metabolites in saliva are indicative of cellular metabolism and disease states [12]. Among its most interesting aspects are extracellular vesicles, such as exosomes, which serve to transport biomolecules associated with tumors and so are of interest in cancer biomarker studies.

Besides ensuring hydration of the mouth, saliva serves several critical functions. It guards tissues, helps in the healing of wounds, supports immune defence, assists digestion, and preserves microbial balance. One of the most significant functions of saliva is to regulate pH levels so as not to lead to acid-induced enamel erosion, dental caries, and other oral disorders. The antimicrobial property of saliva is acquired from compounds such as immunoglobulins (IgA), lysozyme, lactoferrin, and defensins, which synergistically fight against bacterial, viral, and fungal infections [13]. Saliva also performs a significant role in taste and chewing, again establishing its relevance in digestion and oral health.

Saliva is also involved in the regeneration and repair of tissues. Some of the growth factors responsible for cell proliferation, angiogenesis, and wound healing are epidermal growth factor (EGF) and vascular endothelial growth factor (VEGF). The ability to regenerate is an unmistakable pointer towards the therapeutic potential of saliva in the treatment of oral as well as systemic diseases [14]. Because saliva is in constant contact with oral tissues and is also an indicator of overall health, it is a perfect diagnostic vehicle for the detection of disease-associated molecular alterations (Table 2).

**Table 2:** Key salivary biomarkers in OSCC pathogenesis and diagnosis. Biomarkers are categorized by molecular class, with documented concentration changes, functional roles, and clinical applications. References correspond to validated studies in this review. OSCC: oral squamous cell carcinoma, EMT: epithelial-mesenchymal transition, IL-8: interleukin-8, MMP-9: matrix metalloproteinase-9, TP53: tumor protein 53, CDKN2A: cyclin-dependent kinase inhibitor 2A. The arrow (↑) indicates an increased concentration or upregulation of the respective biomarker in OSCC samples compared to healthy controls.

Biomolecule	Specific examples	Concentration in OSCC	Biological role	Clinical utility	References
miRNAs	miR-31, miR-200a, miR-21	↑ 2–5x in early OSCC	Tumor suppression, EMT regulation	Early detection, prognosis monitoring	[1,6,8]
Proteins	IL-8, MMP-9, IL-6	↑ 3–8x (e.g., IL-8: 50 pg/mL → 400 pg/mL)	Inflammation, metastasis promotion	Staging, treatment response	[4,6,15]
Circulating tumor DNA (ctDNA)	TP53 mutations, CDKN2A methylation	Detectable in stage I–II	Tumor-specific genetic alterations	Minimal residual disease monitoring	[5,16]
Metabolites	Lactic acid, phenylalanine	↑ 10x in advanced OSCC	Warburg effect, metabolic reprogramming	Differential diagnosis (vs. benign lesions)	[10]
Exosomes	CD63+ vesicles carrying miR-375	↑ 5x in saliva vs. healthy	Intercellular communication, tumor microenvironment reflection	Liquid biopsy alternative	[4,7]

Saliva as a diagnostic biofluid for oral cancer

Salivary diagnostics has been a revolution in oral cancer detection, and it has several advantages over the conventional methods [5]. Its greatest strength may be that it is not invasive; therefore, sample collection is painless, simple, and extremely patient-friendly [6]. Most of the conventional diagnostic methods, such as biopsies and blood draws, are invasive, expensive, and require specialists. Salivary diagnostics, on the other hand, obviates pain and invasiveness of procedures and thus increasing patient compliance and accessibility of cancer screening.

Another significant benefit of saliva-based diagnostics is its cost and simplicity of sample collection. In contrast to blood samples, saliva samples are easy to collect at home and test without elaborate laboratory facilities. This is a major advantage of saliva-based diagnostics, as it is a viable instrument of mass cancer screening programs in resource-poor settings. Saliva is referred to as the 'mirror of the body' as it reflects the systemic status of the body [16]. Biomolecules from blood, tissue, and cell secretion permeate saliva through passive diffusion, active transport, and exosome transfer. Therefore, saliva contains tumor-specific biomarkers, such as circulating tumor DNA (ctDNA), miRNAs, and proteins, which may be beneficial in comprehending oral cancer initiation and progression. The biomarkers help detect cancer at an early stage, monitor disease progression, and assess treatment response. Omics technologies, including genomics, transcriptomics, proteomics, and metabolomics, provide high-throughput platforms to study large-scale molecular changes associated with OSCC. These approaches enable comprehensive profiling of DNA mutations, RNA expression patterns, protein networks, and metabolic alterations. When combined, these layers form the foundation of ‘salivaomics,’ a multi-dimensional analytical framework that significantly enhances the detection accuracy of salivary biomarkers by capturing the complex biology of tumor development.

Even with these benefits, saliva-based diagnostics is hampered by several issues that need resolving in order to promote its routine clinical use. Among the limitations is the biomarker concentration variability, which is susceptible to influences, such as hydration, circadian variation, food intake, and the method of sample collection. Standardization of saliva sampling and processing methods must be done so that reliable and reproducible results are obtained [10]. Further, sensitivity and specificity issues must be resolved, and this requires the use of more advanced analytical methods, such as sequencing, polymerase chain reaction (PCR), and mass spectrometry, to enhance the detection capability of biomarkers.

Salivaomics: a multi-omic approach to biomarker discovery

Salivaomics has changed biomarker discovery dramatically, and with a detailed multi-dimensional examination of the contents of saliva made available, it enables salivaomics to include genomics, transcriptomics, proteomics, metabolomics, microbiomics, and exosomal science, and provide a broad molecular view of oral cancer. Salivary genomics is aimed at the discovery of DNA-based changes responsible for oral cancer. Tumor suppressor gene mutations like TP53, p16, and MGMT have been widely identified in saliva from oral cancer patients [9]. In addition, loss of heterozygosity and DNA methylation defects are markers for early malignant transformation. ctDNA in saliva provides a non-invasive substitute for tissue biopsies, allowing real-time assessment of tumor burden and disease progression [5]. 

**Figure 1:**
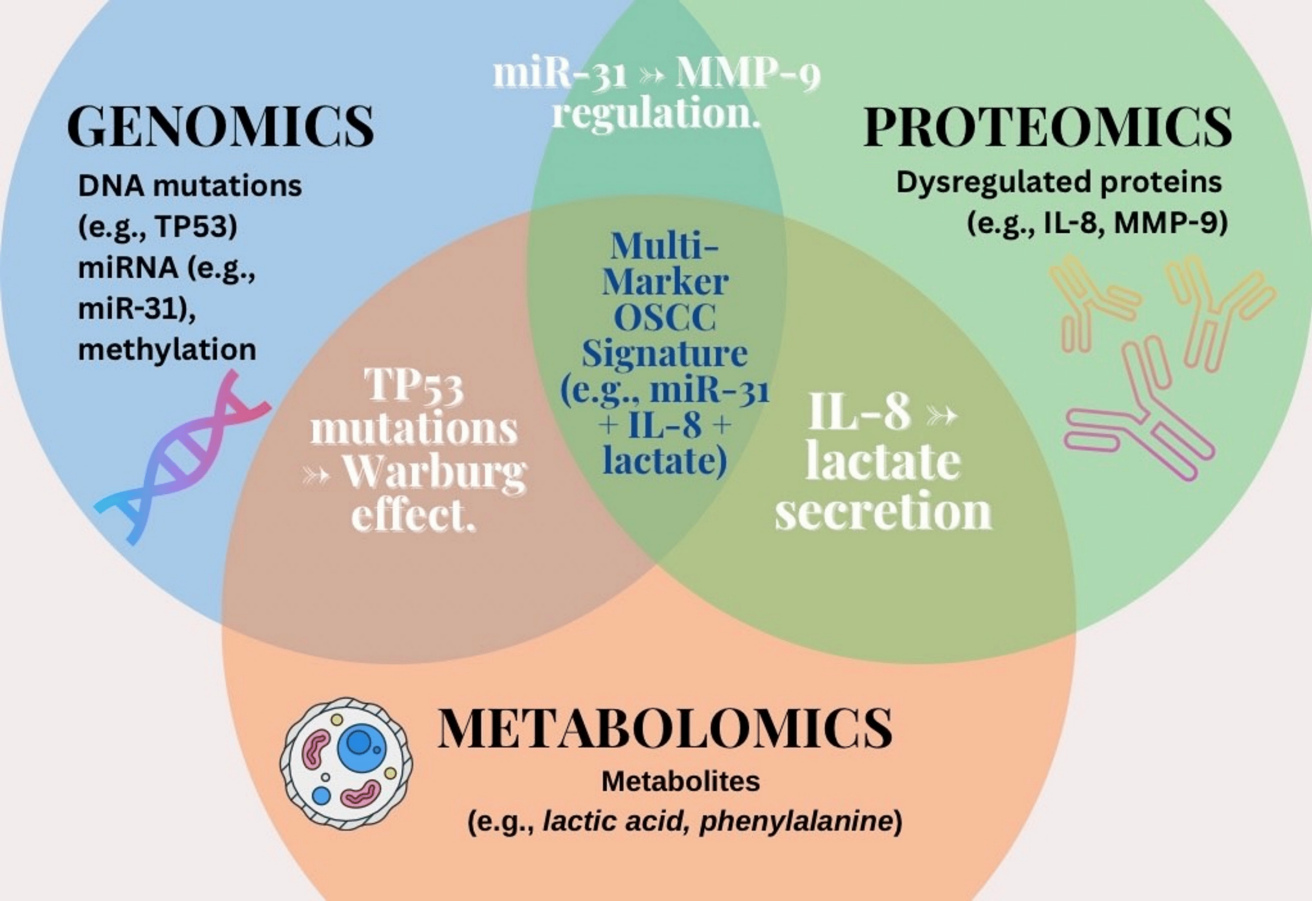
Multi-omics integration in salivary biomarker discovery. Overlapping omics layers (genomics, proteomics, metabolomics) identify high-specificity biomarker panels for OSCC. The center highlights a combined signature (e.g., miR-31 + IL-8 + lactate) with 95% sensitivity. Image Credits: Dr. Anam Khan. Created using Canva (Canva Pty Ltd., Sydney, Australia). OSCC: oral squamous cell carcinoma, IL-8: interleukin-8, MMP-9: matrix metalloproteinase-9, TP53: tumor protein 53.

Salivary transcriptomics identified certain of the mRNA and miRNA biomarkers present in oral cancer. MiRNAs such as miR-146a, miR-205, miR-99a, and miR-100 are specific to target the major oncogenic pathways and potentially serve as primary diagnostic indicators. Salivary proteomics identified dysregulated acute-phase proteins, matrix metalloproteinases (MMPs), cytokines, and oncoproteins that are causative for tumor growth and metastasis. These miRNAs (e.g., miR-31, miR-200a) and proteins (e.g., IL-8, MMP-9) have been validated in large-scale omics studies demonstrating diagnostic sensitivity ranging from 85% to 90% and specificity from 80% to 88% for OSCC detection [17] (Figure 1).

Salivary metabolomics has identified tumor metabolic reprogramming in oral cancer in the form of increased lactic acid, choline, and betaine concentrations, as validated by Panneerselvam et al. [18]. Salivary microbiomics has documented pro-inflammatory pathogen overgrowth, such as *Porphyromonas gingivalis* and *Fusobacterium nucleatum*, implicated in carcinogenesis [6]. Exosomal research has further identified tumor exosomal miRNAs and proteins as a valid and stable biomarker source.

Clinical applications 

**Figure 2:**
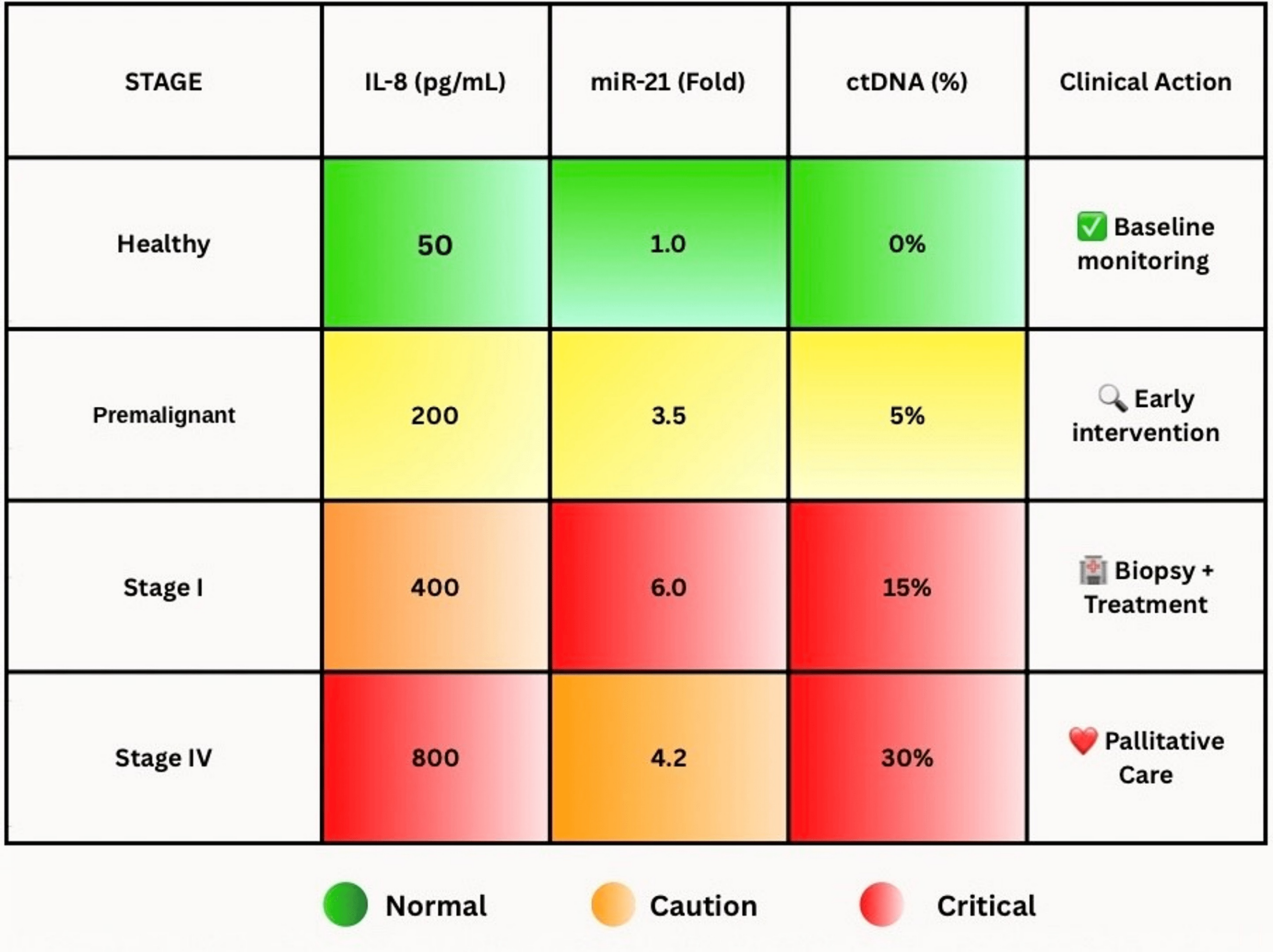
Heatmap with clinical annotations. Clinically actionable salivary biomarker thresholds across OSCC progression stages. Biomarker concentrations (IL-8, miR-21, ctDNA) are categorized by clinical urgency (normal/caution/critical), with corresponding diagnostic or therapeutic interventions. Values reflect mean levels from cohort studies. Image Credits: Dr. Anam Khan. Created using Canva (Canva Pty Ltd., Sydney, Australia). OSCC: oral squamous cell carcinoma, IL-8: interleukin-8, TP53: tumor protein 53, ctDNA: circulating tumor DNA.

The clinical use of salivary biomarkers in the diagnosis of cancer has gone beyond previous visions, providing an easily available, non-invasive, rapid, and cost-effective strategy compared to the existing methods. Biomarkers have come to be critical in the diagnosis of OSCC, the differential diagnosis of premalignant lesions, follow-up for response, prognosis of recurrence, and the detection of even metastatic cancers to the oral cavity [9]. Saliva is both readily accessible and constantly replaced and thus can serve as an outstanding biofluid that can diminish dependency on invasive operations, such as the biopsy. Salivary biomarkers such as proteins (IL-8, IL-6, MMP-9), genetic and epigenetic markers (p53 mutations, hypermethylation of p16 and DAPK, miRNA signatures such as miR-31 and miR-200a), and metabolic profiles (polyamines, lactic acid, phenylalanine) have shown high specificity and sensitivity in distinguishing healthy subjects from OSCC patients in oral cancer detection and screening [7] (Figure 2).

Aside from primary diagnosis, salivary biomarkers also help to differentiate malignant from premalignant lesions like oral leukoplakia and oral lichen planus. Differential biomarker expression of proteins like IL-8, MMP-9, and certain DNA methylation profiles sheds light on the course of these diseases so that intervention may be initiated at an earlier stage [6]. Moreover, machine learning (ML) models combining salivary biomarker panels are also in the process of being constructed to improve predictive value in categorizing high-risk oral lesions [2]. Another important application is in the tracking of disease progression and response to therapy, where tumor response to treatment is monitored through changes in biomarker levels. The reduction of inflammatory cytokines, including IL-1β and TNF-α, by radiotherapy or chemotherapy is a sign of effective treatment, while modified miRNA profiles, including decreases in miR-21 and miR-200c, can be associated with tumor regression. Other proteins, such as EGFR and MMP-9, are used as markers for possible recurrence, allowing for early intervention. The use of wearable biosensors to integrate saliva analysis is a novel development, which enables real-time therapeutic monitoring and minimizes the frequency of invasive testing [19].

Salivary biomarkers have also shown promise in cancer recurrence prediction and prognosis. Increased miR-200c and miR-375 expression after treatment was found to be associated with increased recurrence rates, whereas protein markers such as GRP78 and heat shock proteins (HSPs) show changes that associate with poor prognosis and therapy resistance [20]. ctDNA detection in saliva is a useful method for the monitoring of minimal residual disease, with the advantage of serving as an early warning system for relapse of the tumor. 

The use of AI-based predictive models is being explored to enhance recurrence prediction and personalize post-treatment surveillance plans. Although predominantly investigated in oral cancer, salivary biomarkers are also under investigation for their potential utility in other cancers, including breast, pancreatic, lung, and gastric cancers. Increased saliva levels of HER2, CA15-3, and miR-21 have been found to correlate with the advancement of breast cancer, while changes in pancreatic cancer K-ras mutations and polyamine metabolic signatures indicate saliva as a diagnostic fluid. In lung cancer, miRNA biomarkers, such as miR-21 and miR-486, along with EGFR mutations, have been identified in saliva, highlighting its potential as a useful diagnostic fluid.

These results suggest saliva-based diagnostics to be a potential universal, non-invasive, cancer-screening tool that is not subject to the repeated need for blood collection or invasive biopsy. The ability to monitor continuously disease development and response to therapy by saliva testing is of unparalleled promise for personal medicine. Its extensive clinical usability will depend on rigorous validation tests, standardization of protocols for measuring biomarkers, and official approval to justify reliability and replicability. With the development of biosensing technology, the use of portable, real-time diagnostic devices based on saliva is going to revolutionize cancer detection to make it an accessible, more efficient, and patient-friendly process [21].

Techniques and technologies for biomarker detection in saliva

OSCC is often spotted in later stages, which delays treatment and worsens survival. Standard diagnostics-biopsies, PET/CT scans, or blood labs-are costly, invasive, and demand skilled personnel. Because of these drawbacks, researchers are looking to saliva as a cheaper, gentler way to catch the disease early. Saliva flows past tumors constantly, so it can carry tell-tale molecules before symptoms appear. Gathering it is almost painless, requires no needles, and the samples stay stable without high-tech freezers. Many studies confirm that saliva holds the same critical biomarkers-proteins, DNA, RNA, miRNAs, cytokines, metabolites, and extracellular vesicles-that blood does [22]. Salivary levels of sialic acid, LDH, IL-6, IL-8, TNF-α, VEGF, and MMPs often rise in patients with OSCC. Metabolomic methods, like nuclear magnetic resonance, gas chromatography-mass spectrometry, liquid chromatography-mass spectrometry, and capillary electrophoresis time-of-flight mass spectrometry, will quantify altered metabolites and sometimes utilize and quantify specific metabolites, such as L-phenylalanine and pyruvate, reflecting metabolism, changes associated to cancer metabolic changes, such as the Warburg effect [23].

**Figure 3:**
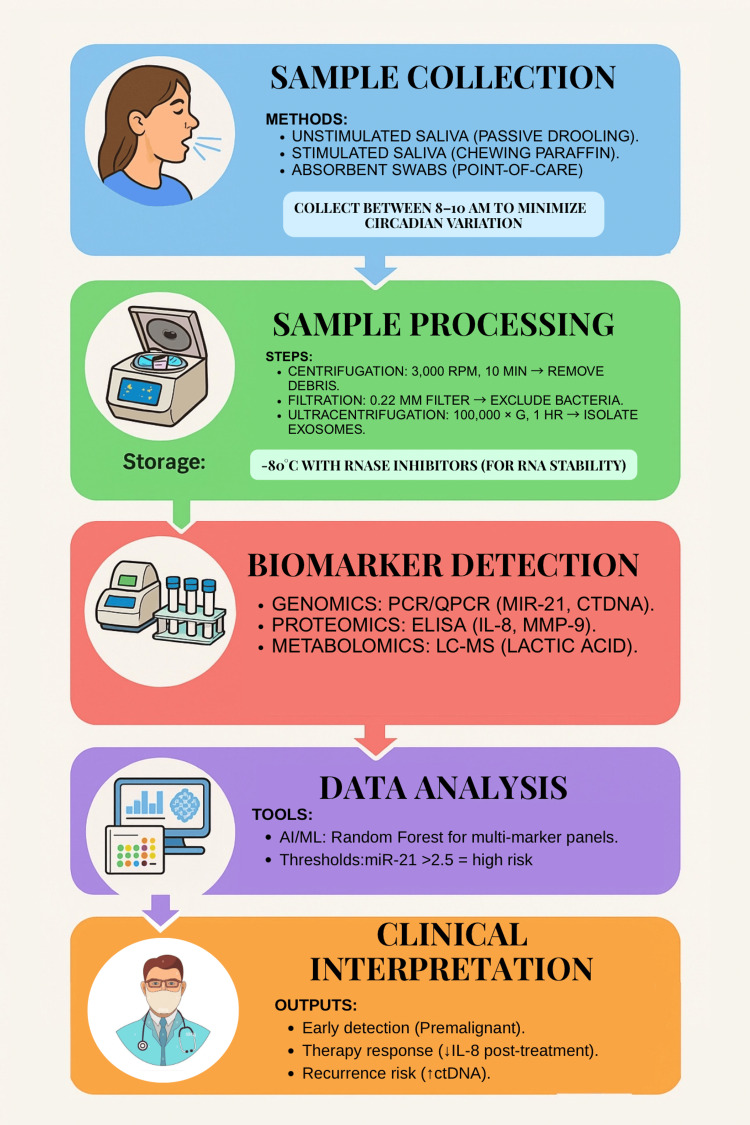
Standardized workflow for salivary biomarker analysis in OSCC detection. From sample collection to clinical interpretation, critical steps include exosome isolation for miRNA stability and AI-driven multi-marker analysis. Protocols follow for reproducibility. Image Credits: Dr. Anam Khan. Created using Canva (Canva Pty Ltd., Sydney, Australia). OSCC: oral squamous cell carcinoma, IL-8: interleukin-8, ctDNA: circulating tumor DNA.

AI and ML can provide incremental improvements in the accuracy of diagnostics by recognizing complicated patterns in biomarker profiles, which they can examine by employing any number of techniques (principal component analysis or cluster analysis) to determine classifications of a patient sample that received a diagnosis [24]. The integration of multi-omics can achieve enhanced sensitivity and specificity for detection in early-stage or personalized OSCC [25]. Standardized workflow for salivary biomarker analysis in OSCC detection. From sample collection to clinical interpretation, critical steps include exosome isolation for miRNA stability and AI-driven multi-marker analysis (Figure 3).

Challenges and limitations 

**Figure 4:**
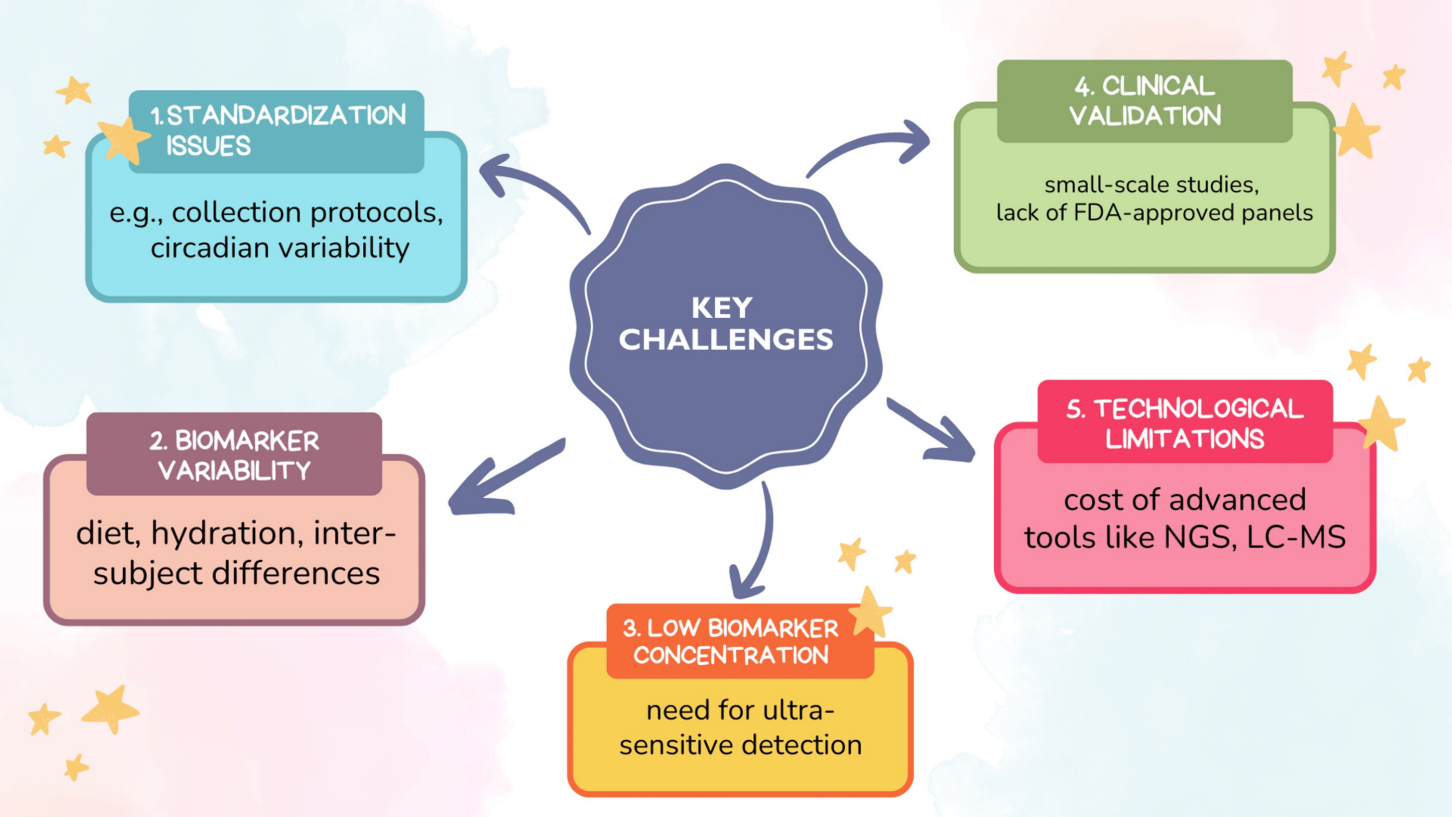
Key challenges in the clinical implementation of salivary biomarkers for oral cancer detection, including standardization, biological variability, and technological limitations. Image Credits: Dr. Anam Khan. Created using Canva (Canva Pty Ltd., Sydney, Australia). NGS: next-generation sequencing; LC-MS: liquid chromatography-mass spectrometry.

Salivary biomarkers provide a non-invasive way to detect oral cancer; however, a number of obstacles limit their use in the clinical sense (Figure 4).

Key challenges include the standardization of sampling and analytical protocols, which is the most pressing issue for clinical implementation, as biomarker variability can result from diet, circadian rhythm, and technical factors. Clinical validation through large-scale, diverse population studies is also a top priority for translation.

Sample Variation & Stability

Saliva is a biological fluid that is easy to collect, but factors such as diet, circadian rhythm, and contamination can affect levels of salivary biomarkers. To reduce variability, the collection must be standardized (e.g., collecting between 8 and 10 am, in a fasting state). Additionally, samples should be processed promptly (i.e., within 30 minutes) and stored at a temperature of −80°C to best preserve the biomarkers. RNA is particularly unstable due to the presence of RNases, so there are commercial stabilizers that preserve RNA to improve sample integrity from the time of collection to analysis [26].

Low Biomarker Concentration

Biomarkers are found at much lower abundance in saliva compared to blood, which limits conventional detection methods, such as enzyme-linked immunosorbent assay and reverse transcription polymerase chain reaction. For increasing our sensitivity of detection, we can use a wide range of advanced methodologies, e.g., mass spectrometry and next-generation sequencing. In addition, our understanding of how biomarkers transfer from blood to saliva is not well understood and raises some additional challenges with validation [27].

Heterogeneity of Cancer

Tumors exhibit genetic heterogeneity such that a biomarker from one patient may not apply to another. There are many possible biomarkers being evaluated that are not strictly cancer-specific, which increases false-positive results. Also, reservoirs of tumor molecules are often located intracellularly, demonstrating the difficulty of finding them in saliva [28].

Biomarker Degradation

Saliva contains enzymes, microbes, and changes in pH that will degrade unwanted proteins and RNA. To make saliva more stable and reliable, we process samples immediately, add enzyme inhibitors, and analyze salivary samples through exosomes [29].

Clinical Translation & Regulation

Many studies utilize smaller, localized samples that lack diversity. For many salivary biomarkers, diagnostic cut-offs are not standardized, limiting clinical application. These regulatory organizations are usually unwilling to approve new tests without having them extensively validated through a large-scale study with multiple sites. Many regulatory organizations will not approve new tests if they cannot manage false positives and negatives [30].

Future Perspectives

New advances in biosensors, nanotechnology, and AI, including ML techniques, will increase the accuracy of the diagnostic test. Multi-omics strategies use various methodologies (i.e., genomics, proteomics, metabolomics, microbiomes) for quality and reproducibility in developing biomarker panels. While there are ongoing challenges in facilities and applications, research will continue as salivary diagnostics move toward clinical implementation [31].

Future directions 

The future of salivary biomarker research in the diagnosis of oral cancer is aimed at the advancement of biomarker discovery, validation, clinical usefulness, and technological development [6]. In order to bring saliva-based diagnostics into the clinic, research must move beyond basic biomarker discovery and address the development of standardized, clinically validated, and highly sensitive multi-biomarker panels that provide reproducible and precise results. A pressing area of research for the future is the identification and categorization of potential oral cancer biomarkers for screening, differential diagnosis, recurrence, prognosis, monitoring of treatment, and detection of metastasis. Current studies have addressed largely early detection, but future research has to also examine biomarkers that anticipate disease progression, responsiveness to therapy, and likelihood of recurrence of cancer. Such biomarkers would be crucial for personalized medicine, allowing individualized treatment regimens based on patients' profiles. In addition, biomarkers reflecting the efficacy of oral cancer treatment must be set up, making real-time evaluation of treatment effect and necessary modification to maximize patients' outcomes feasible. 

To realize such goals, future biomarker research should follow rigorous scientific requirements, including analytic validity, clinical validity, and clinical utility. Analytic validity would ensure the biomarker detection is accurate, reproducible, and sensitive, while clinical validity would determine such biomarkers to be specifically associated with oral cancer and not being confounded by other conditions [32]. Clinical utility must be determined for establishing that biomarkers lead to enhanced patient care by offering information that can be used to make decisions, increasing diagnostic precision, therapeutic choices, and prognosis calculation. Protocols for reporting and biomarker analysis must be standardized to ensure that novel findings are thoroughly validated before they are applied in the clinic [31].

The improvement in technology for detecting salivary biomarkers is another area of significant future research. Since the saliva is full of biomarkers at diluted concentrations compared to blood, ultra-sensitive detection systems have to be engineered to increase the diagnostic efficacy. Biosensors, microfluidic devices, and nanotechnology assays are excellent choices to enhance the sensitivity and specificity of biomarkers. In addition, wearable biosensing devices could revolutionize oral cancer diagnosis by tracking salivary biomarkers in real-time continuously, overcoming the disadvantage of one-point saliva collection and restricting extrinsic factors that are accountable for the variability of biomarkers [2]. 

ML and AI will most probably dominate biomarker discovery, validation, and interpretation. With large-scale biomarker databases, AI-derived models can categorize subtle molecular signatures, and improve accuracy in early detection, as well as reduce false-positive and false-negative rates [6]. Risk stratification and treatment optimization might result from diagnostic computer programs generated by AI that enable more precise individualized patient treatment plans [2].

In addition, multi-omics integration of genomics, proteomics, metabolomics, and microbiomics is an area that needs to be explored in the near future [5]. A single biomarker alone may not be able to provide sufficient diagnostic accuracy; however, a panel of biomarkers evaluating several biological pathways may provide a better estimation of disease status. Multi-omics technologies will facilitate the development of highly sensitive and specific biomarker signatures, as demonstrated by integrated metabolomic and proteomic analyses [10]. Finally, massive clinical trials need to validate salivary biomarkers before implementing them on a large scale in clinics. A majority of the biomarker studies employ small populations of participants and therefore cannot generalize the results. Subsequent research should involve multi-center, population-based studies involving biomarker performance measures across heterogeneous patient populations to establish reliability, reproducibility, and utility in clinical settings (Table 3).

**Table 3:** Roadmap for advancing salivary biomarker diagnostics in oral cancer. Key focus areas, actionable steps, and anticipated outcomes to bridge gaps between research and clinical implementation.

Focus area	Actions needed	Expected outcome
Multi-omics integration	Combine genomics, proteomics, metabolomics	High-specificity biomarker panels
AI & machine learning	Develop predictive algorithms	Improved early detection accuracy
Wearable biosensors	Design real-time saliva analyzers	Continuous monitoring for recurrence
Regulatory approval	Large-scale clinical trials	FDA/EMA-approved diagnostic kits

## Conclusions

Salivary biomarkers represent a paradigm shift in oral cancer diagnostics, offering a non-invasive, cost-effective, and scalable alternative to traditional methods. This review underscores the remarkable potential of salivaomics, encompassing miRNAs, proteins, ctDNA, and metabolites, to enable early detection, real-time monitoring, and personalized treatment of OSCC. Advances in multi-omics technologies, biosensors, and AI-driven analytics have significantly enhanced the sensitivity and specificity of salivary diagnostics, paving the way for chairside and point-of-care applications.

However, the clinical translation of these biomarkers faces challenges, including standardization of sampling protocols, biological variability, and the need for large-scale validation across diverse populations. Addressing these hurdles requires collaborative efforts among researchers, clinicians, and regulatory bodies to establish robust diagnostic guidelines and cut-off values. The future of salivary diagnostics lies in integrated multi-marker panels, wearable devices for continuous monitoring, and AI-powered predictive models. By harnessing these innovations, saliva-based testing could democratize oral cancer screening, particularly in low-resource settings, and usher in an era of precision medicine. As the field evolves, bridging the gap between biomarker discovery and clinical implementation will be critical to reducing the global burden of late-stage OSCC and improving patient outcomes.
